# Composition and In Vitro Effects of Cultivars of *Humulus lupulus* L. Hops on Cholinesterase Activity and Microbial Growth

**DOI:** 10.3390/nu11061377

**Published:** 2019-06-19

**Authors:** Joanna Kobus-Cisowska, Daria Szymanowska-Powałowska, Oskar Szczepaniak, Dominik Kmiecik, Monika Przeor, Anna Gramza-Michałowska, Judyta Cielecka-Piontek, Małgorzata Smuga-Kogut, Piotr Szulc

**Affiliations:** 1 Department of Gastronomical Sciences and Functional Foods, Poznan University of Life Sciences, 60-637 Poznan, Poland; joanna.kobus@up.poznan.pl (J.K.-C.); oskar.szczepaniak@up.poznan.pl (O.S.); dominik.kmiecik@up.poznan.pl (D.K.); monika.przeor@up.poznan.pl (M.P.); anna.gramza@up.poznan.pl (A.G.-M.); 2 Department of Biotechnology and Food Microbiology, Poznan University of Life Sciences, 60-637 Poznan, Poland; 3 Department of Pharmacognosy, Poznan University of Medical Sciences, 61-781 Poznan, Poland; jpiontek@ump.edu.pl; 4 Department of Agrobiotechnology, Koszalin University of Technology, 75-453 Koszalin, Poland; malgorzata.smuga-kogut@tu.koszalin.pl; 5 Department of Agronomy, Poznan University of Life Sciences, 60-621 Poznan, Poland; piotr.szulc@up.poznan.pl

**Keywords:** *Humulus lupulus*, flavonols, antibacterial properties, radical scavenging

## Abstract

Common hop (*Humulus lupulus* L.) has significant health-promoting properties. Hop cones contain resins, essential oils, proteins, polyphenols, lipids, waxes, and cellulose. Hop extracts include bioactive compounds such as polyphenolic compounds (phenolic acids, and flavonols), and chlorophylls. The aim of this study was to compare the pro-health potential of hop cone extracts obtained from three cultivars (Magnum, Lubelski, and Marynka). The results showed that the cones of Magnum cultivar demonstrated the highest biological activity. The sum of phenolic acids and flavonols in ethanol extract was the highest for this variety and was equal 4903.5 µg/g dw. Ethanol extracts of Magnum cultivars showed the highest degree of iron ion chelation (55.43–88.76%) as well as the activity against 1,1-diphenyl-2-picrylhydrazyl radical (4.75 mmol Tx/g dw). Hop cone extracts as cholinesterase inhibitors showed high potential for aqueous variants. In terms of antimicrobial activity, all investigated extracts demonstrated strong inhibition against *Staphylococcus aureus* and *Staphylococcus epidermidis*, with the Magnum cultivar showing the strongest inhibition. Owing to the biofunctional features of hop cone, it can be concluded that it is an attractive raw material with pro-health potential that can be used much more widely in food technology. However, it should be noted that toxicological tests and in vitro tests must be carried out before the raw material is used in food production.

## 1. Introduction

Common hop (*Humulus lupulus* L.) is a climbing plant belonging to the Cannabaceae (hemp) family and has been included in the order Rosales (rosids) since 2003. For many years, only the species *Humulus lupulus* (common hops) and *Humulus japonicus* (Japanese hops) belonged to the genus *Humulus*. The species *Humulus yunnanensis* originating in the southern Chinese province of Yunnan, was described for the first time in 1936; previously it was identified as *Humulus lupulus* [1,2]. Hops are now widely grown in all temperate regions of the world [3,4]. Hop cones contain, inter alia, resins, essential oils, proteins, polyphenols, lipids, waxes, and cellulose. Hop resins constitute 10–30% of the dry matter of the cone. They are divided into soft resins (9.0–27.5% of cone dry mass) and hard resins (1.5–2.5% of cone dry mass). Soft resins include acids responsible for imparting the bitter taste in beer. Bitter acids present in hops contain the prenylated phloroglucinol derivatives humulons (α-acids) and lupulons (β-acids) [5,6]. Like hop resins, essential oils are secondary metabolites of hops that are secreted by the lupulin glands. The majority of the essential oils consists of hydrocarbons (70%) and an oxygen fraction (30%). Hydrocarbons can be classified into three groups: aliphatic hydrocarbons, monoterpenes, and sesquiterpenes. Myrcene is the most important monoterpene. Sesquiterpenes with the highest concentration are caryophyllene, humulene, and farnesene. Oxygen compounds present in hop oil include terpenic alcohols, dominated by linalool and geraniol, oxidized sesquiterpenes, as well as other alcohols, oxides, and esters [1,3].

Another important group of compounds present in hop cones are polyphenols, which constitute 3–6% of the dry matter of the cone. The most important polyphenols of hops include prenylated chalcones (xanthohumol, desmethylxanthohumol), from which isomeric flavanones (isoxanthohumol, 8-prenylnaringenin) are formed. Hop flavonoids are mainly composed of chalcones having isoprenyl or geranyl groups. Xanthohumol is the most important prenylated chalconium in terms of concentration and biological activity. It constitutes 80–90% of all prenylated flavonoids, i.e., from 0.1% to more than 1.7% of the dry matter of cones, depending on the cultivar [7,8].

The cones of female hop plants are mainly used as an ingredient for the brewing industry [2]. There are reports in the literature about the possibility of hops being used as an ingredient in tea. The literature data indicate that infusions from hop cones have calming, diuretic, analgesic, and strengthening effects, as well as anti-inflammatory and antibacterial effects [9]. However, there are no reports in the literature on the possibility of hop cone being a source of biologically active compounds that can be used in the prevention of neurodegenerative diseases.

The aim of this study was to evaluate the pro-health potential of hop cone extracts obtained from three cultivars (Magnum, Lubelski, and Marynka) to act as acetylcholinesterase (AChE) and butyrylcholinesterase (BChE) inhibitors, and as a source of substances with antimicrobial activity.

## 2. Materials and Methods

### 2.1. Material

Cones of common hops (*Humulus lupulus* L.) of the cultivars Magnum, Lubelski, and Marynka were used in the study. The hop plantation is located in Malice (Kujawsko-Pomorskie province, Kcynia commune: 52°59′52.6′′ N 17°31′20.6′′ E). Depending on the cultivar, the cones were harvested at the turn of August and September 2017. After harvesting, the cones were dried using the freeze-drying method. The raw material was characterized by taking into account the basic chemical composition for the cultivars Magnum, Lubelski, and Marynka, respectively (34.83 g/100 g, 34.21 g/100 g, 35.18 g/100 g lipids; 4.52 g/100 g 4.43 g/100 g, 4.56 g/100 g ash; 19.41 g/100 g, 19.46 g/100 g, 19.34 g/100 g protein; 23.41 g/100 g, 23.47 g/100 g, 23.29 g/100 g dietary fiber; 17.42 g/100 g, 17.55 g/100 g, and 17.37 g/100 g other carbohydrates). The cones were extracted after grinding in a Grindomix GM 200 (Retsch, Haan, Germany) for 180 s at 1792× *g* at 21 °C.

### 2.2. Extraction

The cones were subjected to water and ethanol-water extraction. The water extract was obtained using a three step extraction method. At 85 °C, 1000 mL of water (400 mL, 300 mL, and 300 mL, respectively) was mixed with 50 g of raw material and extracted for three times, each time for 10 min. The extracts were filtered and centrifuged (815× *g*, 15 min) each time. The fractions were decanted and filtered (Whatman 1:11 μm), and the obtained supernatants were combined and freeze-dried. Ethanol-water extract (40%) was obtained by mixing 50 g of cones with 400 mL of solvent. The samples were shaken in a water bath for 15 min at 21 °C at constant amplitude, and the supernatant was decanted. In the second and third extraction stages, the sample of crushed hop cones was again mixed with 300 mL of solvent. The solutions from the three stages were combined and filtered (Whatman 1:11 μm), and the final extract was obtained. The total time of the extraction was 45 min. The obtained supernatants were combined; the solvent was evaporated on a vacuum rotary evaporator and the water residue was freeze-dried.

### 2.3. Composition of Chlorophylls and Carotenoids

The content of carotenoids and chlorophyll dyes was determined using the method based on spectrophotometric determination of absorbance at the following wavelengths: 662 nm, 644 nm, and 440 nm [10,11]. The concentrations (mg/L) of chlorophyll A and B were determined by measuring the absorbance of the tested extracts at 662 and 644 nm (A_662_ and A_644_, respectively), according to the following equations:

Chlorophyll A = 9.784 * A_662_ − 0.99 * A_644_, (1)

Chlorophyll B = 21.426 * A_644_ − 4.65 * A_662_, (2)

Total carotenoids concentration (mg/L) was calculated using the absorbance of tested extracts at 440 nm (A_440_) and concentration values for chlorophyll A (C_a_) and chlorophyll B (C_b_):

Total carotenoids = 4.695 * A_440_ − 0.369(C_a_ + C_b_), (3)

Then, the obtained results were given in mg/g of dry product.

### 2.4. Content of Flavonols and Phenolic Acids

The procedure was based on the method published by Kobus et al. [12]. Phenolic acids and flavonols were analyzed using Agilent UPLC equipped with a Bin Pump Infinity DAD 1290 detector (for phenolics acids λ = 260 nm and 310 nm and for flavonols λ = 370 nm). Separated compounds of phenolic acids and individual flavonols were determined as chlorogenic acid, ferulic acid, vanillic acid, gallic acid, *o*-coumaric acid, *p*-coumaric acid, cinnamic acid, syringic acid, *p*-hydroxybenzoic acid, caffeic acid, catechin, epicatechin, quercetin, rutin, and kaempferitrin. Compounds were identified using the standards dissolved in methanol.

The single-reference method was applied to determine a relationship between peak area and the concentration of the analyzed standard.

### 2.5. Chelating Activity

Chelating activities of crude asparagus extracts were measured according to the method by Kobus-Cisowska et al. [13]. In the colorimetric assay, the amount of unchelated Fe^2+^ was determined in crude extract after its reaction with 3-(2-pyridyl)-5,6-bis(4-phenylsulfonic acid)-1,2,4-triazine monosodium salt (i.e., ferrozine). One milliliter of sample, 0.1 mL of 2 mM FeCl_2_ and 0.2 mL of ferrozine reagent were added to each tube. The mixture was vortexed for 60 s and left for 20 min at room temperature. Absorbance values were recorded (*λ* = 562 nm) using the Meterek SP 830 apparatus (Taiwan). Deionized water as a control and ferrozine as a reference sample were used. The chelating activity was calculated as a percentage.

### 2.6. Antiradical Activity with DPPH and ABTS

The principle of the method was based on spectrophotometric (Metertech SP-830, Taipei, Taiwan) measurement of reaction mixture color at 517 nm wavelength. In this method, depending on the antioxidant capacity of the extract, 1,1-diphenyl-2-picrylhydrazyl (DPPH) free radicals were scavenged [14]. Antioxidant activity was expressed in mmol Trolox per 1 g of extract dry matter and as EC50 (mg extract/mL). EC50 value was the concentration of the sample required to inhibit 50% of DPPH free radicals. Negative control contained deionized water. The absorbance value for the negative control was subtracted from the absorbance value for tested extracts. Activity toward 2,2′azino-bis(3-ethylbenzothiazoline-6-sulphonic acid (ABTS) cation radicals was determined using the method described by Re et al. [15]. The results were given as Trolox equivalents (Tx) using the calibration curve.

Analysis of antioxidative activity was performed for tested hop samples and for standards of compounds present in *Humulus lupulus* (i.e., chlorogenic acid, quercetin) dissolved in aqueous-methanol (3:1, *v*/*v*) solvent.

### 2.7. Cholinesterase Inhibition

The modified spectrometric method developed by Ellman et al. [16], which was described by Kobus-Cisowska et al. [17], was used to measure the activity of the extracts as AChE and BChE inhibitors. A POLARstar Omega (BMG LABTECH, Germany) plate reader was used for measurements of 96-well plates of the maximum volume of 300 μL. The hydrolysis of acetylthiocholine/butyrylthiocholine caused a color change. The absorbance of the enzymes was measured at a wavelength of 412 nm, ten minutes after pipetting on a microplate. The reaction mixture containing 0.1 mL of 0.3 mM 5,5-dithio-bis-(2-nitrobenzoic acid) (DTNB, Sigma Aldrich, Germany), 10 mM NaCl and 2 mM MgCl_2_•6H_2_O solution, 0.575 mL 50 mM Tris-HCl buffer (pH = 8.0), 25 μL of 0.28 units/mL AChE/BChE (Sigma Aldrich, Germany), and 0.2 mL of tested extract was tested at wavelength 405 nm and at temperature 22 °C. The measurement was conducted after 20 min (BChE) or 60 min (AChE) after adding all ingredients into a microplate. Blank sample contained Tris-HCl buffer instead of tested extract. A positive-negative control was applied and it consisted of 90.7 μM eserine instead of tested extract. All samples were analyzed in eight independent repeats. The inhibitory activity of each enzyme was calculated with the use of a calibration curve. The calibration curves were prepared using serine as a standard at concentration ranges between 0.09–6.10 μM for AChE and 0.09–8.57 μM for BChE.

### 2.8. Antimicrobial Activity of Hop Cones with Respect to Potentially Pathogenic Microorganisms

Indicator microorganisms were transferred to test tubes containing Mueller-Hinton medium. They were cultured at 37 °C for 24 h. Next, liquefied agar medium was inoculated with 10% (*v*/*v*) 24-h indicator culture and poured into Petri dishes to obtain a distinct confluent layer. After solidification of the broth medium inoculated with the indicator microorganisms, wells were made with a cork borer. Each well was supplemented with 150 µL of liquid extract from hops or suspension of ground hop cone medium. Next, the diameters of the growth inhibition or reduction zone of indicator bacteria were measured. The inhibition of the growth of the indicator microorganism was manifested by complete lightening around the place where the liquid extract or slime was transferred. It indicated bactericidal activity of the bacterial strain. Bacteriostatic properties were determined by measuring the diameter of the growth inhibition zone (indicator strain growth limitation).

### 2.9. Statistical Analysis

Data were analyzed statistically by STATISTICA^TM^ PL 13.1 (StatSoft, Kraków, Poland). Individual parameters were described statistically. The results presented in the article are the arithmetic mean of at least two series replicated three times. The mean values of the traits under study were compared by analysis of variance for factor systems with a diversified number of observations. Differences between the groups were assessed by Tukey’s test or Spjotvoll’s test (extended Tukey’s test for samples of unequal sizes). The assumptions of the analysis were also checked. Pearson correlation coefficients were calculated to assess the strength of relation between the samples under analysis. The significance of the correlation coefficient was checked by means of Student’s *t*-test. The significance of statistical inference was *p* < 0.05.

## 3. Results

### 3.1. Chlorophyll and Carotenoids

The content of chlorophylls and carotenoids was significantly differentiated in the examined hop cones and depended on the solvent used (Table 1). Higher concentrations of total chlorophylls and carotenoids were found in ethanol-water extracts, whereas lower concentrations were found in water extracts. The highest sum of chlorophylls (1.99 mg/g dw) was determined in water-ethanol excavator for cones of Marynka cultivar (MaE), and the lowest sum was found in water extract of Lubelski cultivar (LW) and Marynka cones (MaW), 0.48 mg/g dried weight (dw), and 0.49 mg/g dw, respectively. The highest level of chlorophyll α was found in MaE extract (1.32 mg/g dw) (70% of the sum of all chlorophylls), and the lowest level was found in LW (0.37 mg/g dw). Statistical analysis showed that chlorophyll content significantly depends on the cultivar of the hop cone and extraction efficiency on the solvent used (*p* < 0.05).

### 3.2. Phenolic Acids and Flavonols

The highest content of phenolic acids and flavonols in both ethanol and water extracts was found in the extracts from the cones of Magnum cultivar. The higher sum of phenolic acids and flavonols was found in ethanol extracts (3083.9–4903.5 µg/g dw), compared to water extracts (2466.9–4049.2 µg/g dw; Table 2). The dominant phenolic acid in the analyzed extracts was chlorogenic acid, which ranged from 191.4 µg/g (LW) to 1077.0 µg/g (ME). A high content of *o*-coumaric, *p*-coumaric, cinnamic, and syringic acids was also found. Among flavonols, epicatechin and rutin were dominant; and significant amounts of quercetin and kaempferol were also found.

### 3.3. Chelating Activity

**Figure 1:**
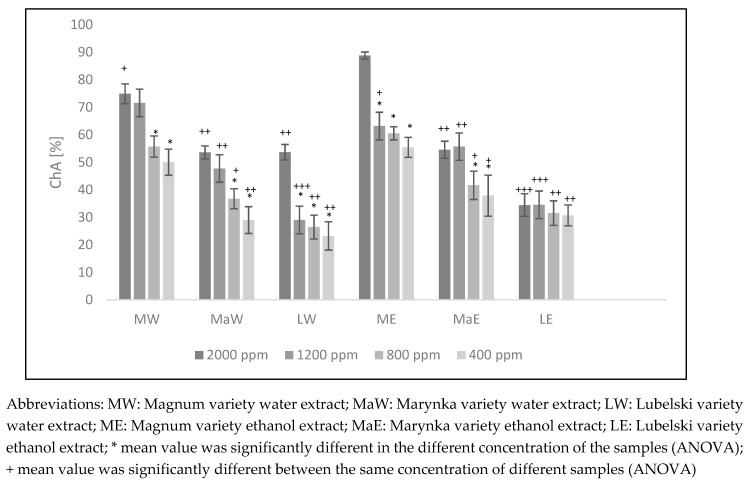
Chelating activity of extracts of *Humulus lupulus* hops.

Among the examined hop cone extracts, the highest amount of iron ions (55.43–88.76%) was chelated by the ethanol extracts of the Magnum cultivar. Among the tested extracts of hops, a lower metal chelating ability was demonstrated for water extracts, and the extract of LW had the lowest chelating ability. It was also found that an increase in the concentration of extracts resulted in a 2.3-fold increase in the chelating activity of LW extract, whereas the chelating activity for LE, MaE, and MW extracts increased by 1.2-, 1.4-, and 1.5-fold, respectively. (Figure 1).

A statistically significant linear correlation was found (Table 3) between chelating activity of analyzed hop cone extracts and the content of ferulic acid > epicatechin > syringic acid > *p*-coumaric acid.

### 3.4. Antioxidant Activity DPPH and ABTS

All analyzed extracts exhibited DPPH radical scavenging activity (Table 4). Water extracts of all cultivars were characterized by the highest activity toward radical, measured in Trolox equivalents and amounted to 4.75 mmol Tx/g dw for Magnum cultivar, 4.37 for MaE, and 4.11 mmol Tx/g dw for LW. Quantities of extracts from hop cones allowing to obtain 50% of DPPH radical inactivation capacity ranged from 0.31 mmol Tx/g dw for LW extract to 0.98 mmol Tx/g dw for LE extract and were 20–60% lower for water extracts compared to ethanol ones. In the ABTS cation radical method, contrary to the DPPH method, it was demonstrated that ethanol extracts from hop cones scavenged the radicals to a greater extent than water extracts, on an average by 60–90%. Antioxidant activity ranged from 1.32 mmol Tx/g dw for Magnum cultivar to 2.43 for Marynka. No statistically significant differences in the activity of extracts depending on the cultivar of the examined hop cones were found.

A statistically significant linear correlation was found between the activity of the analyzed extracts from hops in relation to DPPH and their content of gallic acid > *p*-hydroxybenzoic acid > epicatechin, and between the activity in relation to ABTS and the content of chlorogenic acid > quercetin > gallic acid > catechin.

### 3.5. Cholinesterases

**Figure 2:**
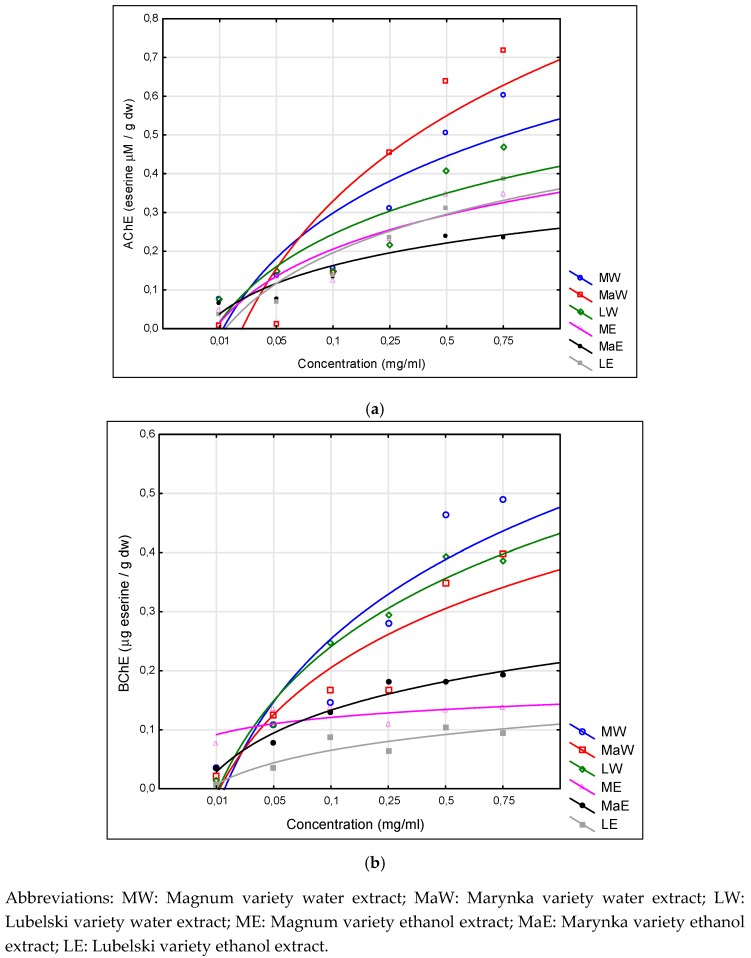
Activity of extracts of *Humulus lupulus* hops as acetylcholinesterase (**a**) and butyrylcholinesterase (**b**) inhibitors.

The examined extracts from hop cones were characterized in terms of inhibition of cholinesterase (ChE) activity—AChE and BChE. Inhibiting capacity was expressed in eserine equivalents (Figure 2).

All tested extracts from hop cones showed ChE inhibition activity in a concentration-dependent manner. The results showed that the extracts prepared from the cones of all hop cultivars demonstrated higher activity as AChE inhibitors than BChE inhibitors (Figure 2). It was found that water extracts were characterized by higher activity against AChE and this activity decreased in the order of MaW > MW > LW. Another relationship due to cultivar was found in the case of the evaluation of the activity of both water and ethanol extracts against BChE, where the extracts prepared from cones of Magnum cultivar demonstrated the highest inhibitory activity, and this activity was as follows: MW > LW > MaW. There was no relationship between the activity with respect to examined ChE and the hop cone cultivar. Ethanol extracts inhibited AChE in the order of ME > LE > MaW, while inhibited BChE in the order of MaE > ME > LE.

The activity of ethanol extracts against ChE increased with increasing concentration. A statistically significant linear correlation was demonstrated (Table 4) between the inhibition capacity of AChE and their content of *o*-coumaric acid > epicatechin > kaempferol > vanillic acid and between the inhibition capacity of BChE and the content of p-hydroxybenzoic acid > rutin > chlorogenic acid > quercetin.

The study concluded that water extraction affected the activity of hop cone extracts more as ChE inhibitors. However, there was no relationship between the activity and the hop cultivar.

### 3.6. Antimicrobial Activity

The well-diffusion method was used to demonstrate antimicrobial activity of hop cones. Water extracts and homogeneous mixture of ground cones suspended in saline solution were studied. Reference strains and clinical isolates (Table 5) were tested. The highest activity for water extracts of all tested cultivars was found with respect to *Staphylococcus aureus* ATCC 25923, and the value for Magnum cultivar was 39 mm, while lower activity was found for a strain of clinical origin (28 mm). Similarly, high antimicrobial activity was observed for *Staphylococcus epidermidis* ATCC 12228 (34 mm) and it was also lower with respect to clinical strain (25 mm). The weakest antibacterial potential of water extracts was demonstrated against a member of the family Enterobacteriaceae, *Enterococcus faecium*. The size of the growth inhibition zone did not exceed 5 mm for both the reference strain and the clinical isolate. The weakest antimicrobial potential was demonstrated for MaE. In this case, the largest growth inhibition zone was shown for *S. aureus* ATCC 25923, which was 18 mm. Moreover, the study showed that water extracts are characterized by significantly higher antimicrobial activity compared to ground cones suspended in physiological saline solution. This relationship was visible for all the three hop cultivars.

The brightness zone around the well with a suspension of ground cones of the Magnum cultivar in relation to *S. aureus* did not exceed 8 mm for the reference strain and 4 mm for the clinical strain. However, for *Propionibacterium acnes* and *E. faecium* species, no antimicrobial activity was demonstrated for both reference strains and clinical isolates.

## 4. Discussion

The properties of hop cone extracts result from the content of bioactive ingredients that determine the antioxidant characteristics and preventative activity against enzymes responsible for the development of civilization diseases, including neurodegenerative ones. In many studies, hop was compared to hemp (*Cannabis sativa*). Hop and hemp properties are similar, as they belong to the same biological family—*Cannabaceae*. Apart from sedative and soporific properties, hop and hemp may play another significant therapeutic role. Studies show that both plants possess rich antibacterial, antioxidative and neuroprotective properties [18,19]. The activity of chlorophylls in radical chain reactions is repeatedly emphasized in the literature. Owing to the presence of double coupled bonds, chlorophylls are effective photoreceptors and photosensitizers [20,21,22]. Strong absorption of visible radiation causes their energetic status stimulation, and excess energy is transferred to an oxygen molecule, which can initiate oxidation processes, thereby proving a pro-oxidative effect [23,24]. The presence of carotenoids in hop cone extracts, which are characterized by high antioxidant and biological activity in biological systems may determine the antioxidant activity of ethanol extracts of the examined hop cones [25]. As shown in the work, the content of these compounds depended on the cultivar, and the higher content of chlorophylls was not always correlated with a higher content of carotenoids. The tested extracts from hop cones were characterized by polyphenol content depending on cultivar and extraction conditions. A raw material cultivar determines the composition and its properties only in the context of antioxidant activity but also of health-promoting effects. Polyphenols are responsible not only for taste and color, but also for pro-health effects, including antioxidant activity. This activity results from the specific structure of polyphenols and the presence of OH hydroxyl groups [26,27]. A common feature of polyphenols, resulting from their characteristic structure, is the ease with which they join the redox reaction [28,29]. This especially applies to those compounds in which functional hydroxyl groups occur in the *ortho*- and *para*-position. It was found that such phenols have a much lower oxidation potential than mixtures with *meta*-diphenols [26,29]. It was proven that flavonoid content determines the antioxidative capacity of extracts [17,28,30]. This activity primarily includes the ability to inactivate the free radicals, e.g., the peroxide anion radical, hydroxyl radical, singlet oxygen, and lipid radicals [31]. In this work, a positive correlation was found between individual compounds in *Humulus* hops and activity measured by radical scavenging tests.

The data in literature points out that the antioxidative capacity is influenced by numerous agrotechnical factors but also by the cultivar. The way to determine the antioxidant potential of plant preparations is determination of their metal chelating efficiency. Excessive accumulation of metal ions leads to oxidative stress due to increased formation of reactive oxygen species. These processes are one of the factors leading to cardiovascular diseases, cancers, diabetes, atherosclerosis, and neurological disorders. Antioxidants bind to metal ions and stabilize them [32,33]. As shown in this study, hop cones are rich in polyphenolic compounds (Table 2). Differences in the content of ethanol and water extracts may explain the higher chelating activity of metals by ethanol extracts compared to water extracts. Another important marker used to evaluate the pro-health character of plant material is its antiradical property. According to Inui et al. [34], the highest percentage of antioxidant capacity, determined with the DPPH radical method, among compounds present in hop cones was demonstrated for (−)epigallocatechin, procyanidin B_2_, and quercetin at a concentration of 12.5 µmol/L. Abram et al. [2] analyzed the antioxidant properties of hop cones and leaves and found that the inhibitory concentration (IC_50_) of the DPPH radical was lower for hop cones than for leaves. According to Wang et al. [35], the concentration of hop polyphenols at 0.025 mg/mL completely deactivated free radical DPPH, which is consistent with the results obtained in this study. In the case of ability to deactivate DPPH free radicals, higher activity was demonstrated for water extracts, which most probably results from hydrophilic properties of the radical itself. Nafis et al. [36] showed that *C. sativa* DPPH radical scavenging potency (EC50) approximates 1.6 mg/mL), which is a four-fold lower value than our results noted for aqueous hop extracts. (Table 3). However, Onder et al. [37] showed that DPPH free radical was most effectively inhibited by hop extracts in which n-hexane was the solvent compared to acetone, methanol, ethanol, and water extracts. Moreover, hop extracts show antiradical activity against the ABTS radical, which has been experimentally confirmed in this study. As in the present study, Wojdyło et al. [38] demonstrated that water extracts from hop cones deactivated the DPPH radical better than ABTS. Inui et al. [34] showed that the functional properties of hops depend on different polyphenols, their proportions, and synergistic effect. As indicated in previous work, the hop variant has an effect on the concentration of the bioactive compounds and, therefore, the varied results of the antioxidant activity result directly from the hops cultivar.

A novelty in terms of evaluation of hop cone biofunctionality is the determination of a marker related to the activity of AChE and BChE inhibitors used in the treatment of neurodegenerative diseases, especially in Alzheimer’s disease [39,40]. The use of pharmaceutical preparations available in the market that inhibit ChE activity is associated with the occurrence of many troublesome side effects. Currently, inhibitory properties with respect to AChE and BChE are attributed to natural compounds of plant origin [39,41]. Damage to neurons by reactive oxygen species is one of the main causes of Alzheimer’s disease. Currently, only symptomatic treatment is used through the use of AChE and BChE inhibitors. Oberbauer et al. [42] conducted a study to evaluate the prevention of neurodegeneration by prenylflavonoid derivatives from hops. Their study showed that prenylflavonoid derivatives from hops have strong neuroregenerative and neuroprotective properties in the treatment of brain, spinal cord damage, and Alzheimer’s disease. In another study on neuronal activity of xanthohumol, young and old mice were fed a diet containing prenylflavonoid for eight weeks. One of the hypotheses of neurodegenerative disease formation is damage to neurons by aluminum. Gonzalez-Munoz [43] et al. assessed an effect of mouse dietary supplementation with beer on aluminum neurotoxicity. According to researchers, beer was effective in protecting against neurotoxic effects of aluminum. The authors suggest that these properties are related to hop content in beer. In vitro and in vivo studies have shown that bioactive plant polyphenols bind to toxic amyloid in the cerebral cortex and prevent its deposition. Compounds with such properties include caffeic acid, catechin, epicatechin and epigallocatechin gallate, curcumin, ferulic acid, gallic acid, kaempferol, myricetin quercetin, resveratrol, rutin, and rosmarinic acid [44,45]. These compounds are found in hop extracts, and hence it can be assumed that they affect the AChE and BChE inhibiting activity. The results of this study indicate that the tested hop samples have inhibitory activity against the tested enzymes. Differences in the obtained results confirm that not only the type of extractant used determines this activity. First of all, the activity depends on the cultivars of hops. Another important marker used in the evaluation of raw materials in terms of their biofunctionality is antimicrobial activity, especially against pathogenic microorganisms. The results at work indicate that the hop cultivar is important for the level of antimicrobial activity. Antimicrobial properties of active compounds found in hop cones are often associated with the presence of xanthohumol, the main prenylated flavonoid present in hops [46,47,48]. Mizobuchi and Sato [49] studied prenylated hop flavonoids for their ability to inhibit the growth of gram-positive bacteria and molds. Xanthohumol and 6-prenylnaringenin had the strongest antibacterial properties in relation to *S. aureus*. Similar observations were made in this study. The authors concluded that the presence of the prenyl group may favor flavonoid transport into the cell, or facilitates the blocking of enzymes by binding in the active center. Xanthohumol and lupulone showed the strongest antibacterial properties among the examined compounds. It can be concluded that the extraction of active compounds takes place under the influence of increased temperature, which is clear from the results obtained. Water extraction at an elevated temperature allowed the release of active compounds showing antimicrobial properties, as opposed to the suspension obtained by grinding dried material and suspending it in NaCl solution.

## 5. Conclusions

The results of this study showed an interesting pro-health potential of the cones of three different cultivars of hops. This potential is closely related to the content of several biofunctional compounds present in them. However, the activity of hop cone extracts as a ChE inhibitor is of value, which can be used in the treatment and prevention of neurodegenerative diseases. In light of our study, we can conclude that hop cone extracts have a much wider potential for their use in food technology than just brewing.

## Figures and Tables

**Table 1:** The content of chlorophyll and carotenoids in *Humulus lupulus* L. hops extracts.

Extract	Chlorophyll α [mg/g dw]			Chlorophyll β [mg/g dw]			Total Chlorophyll [mg/g dw]			Carotenoids [mg/g dw]		
MW	0.44 ^b^	±	0.02	0.10 ^a^	±	0.03	0.54 ^a^	±	0.03	0.59 ^b^	±	0.03
MaW	0.38 ^a^	±	0.02	0.11 ^a^	±	0.02	0.49 ^a^	±	0.06	0.46 ^a^	±	0.01
LW	0.37 ^a^	±	0.05	0.11 ^a^	±	0.03	0.48 ^a^	±	0.11	0.46 ^a^	±	0.02
ME	1.32 ^d^	±	0.01	0.56 ^b^	±	0.01	1.88 ^b^	±	0.09	1.11 ^c^	±	0.05
MaE	1.33 ^d^	±	0.03	0.66 ^b^	±	0.01	1.99 ^b^	±	0.08	1.31 ^d^	±	0.09
LE	1.07 ^c^	±	0.04	0.76 ^b^	±	0.02	1.83 ^b^	±	0.09	1.22 ^d^	±	0.06

The mean values in the column marked with different small letters indicate the significance of differences (*p* ≤ 0.05). Abbreviations: dw, dried weight; MW, Magnum variety water extract; MaW, Marynka variety water extract; LW, Lubelski variety water extract; ME, Magnum variety ethanol extract; MaE, Marynka variety ethanol extract; LE, Lubelski variety ethanol extract.

**Table 2:** Polyphenols content in *Humulus lupulus* L. hops extracts.

Polyphenol [µg/g dw of Extract]	MW	MaW	LW	ME	MaE	LE
Chlorogenic acid	903.3 ^b^ ± 9.8	126.6 ^a^ ± 1.1	191.4 ^a^ ± 1.4	1077.0 ^c^ ± 7.0	133.7 ^a^ ± 0.6	734.1 ^b^ ± 17.7
Ferulic acid	0.8 ^a^ ± 0.0	0.5 ^a^ ± 0.0	0.7 ^a^ ± 0.0	1.4 ^a^ ± 0.0	0.4 ^a^ ± 0.0	0.9 ^a^ ± 0.0
Vanillic acid	5.9 ^a^ ± 0.0	4.6 ^a^ ± 0.1	2.4 ^a^ ± 0.0	13.3 ^c^ ± 0.2	9.3 ^b^ ± 0.0	8.2 ^b^ ± 0.2
Gallic acid	17.3 ^b^ ± 0.2	10.6 ^a^ ± 0.3	15.4 ^b^ ± 0.2	30.6 ^c^ ± 0.4	18.7 ^b^ ± 0.1	18.8 ^b^ ± 0.5
*o*-coumaric acid	33.9 ^a^ ± 0.1	67.8 ^b^ ± 0.3	23.0 ^a^ ± 0.2	66.5 ^b^ ± 0.1	128.3 ^c^ ± 0.5	37.9 ^ab^ ± 0.4
*p*-coumaric acid	56.2 ^a^ ± 0.2	89.2 ^b^ ± 0.3	44.5 ^a^ ± 0.2	88.0 ^a^ ±0.1	256.5 ^c^ ± 1.0	69.4 ^a^ ± 0.4
Cinnamic acid	6.2 ^a^ ± 0.0	110.7 ^d^ ± 0.3	66.0 ^c^ ± 0.2	109.5 ^d^ ± 0.1	31.0 ^b^ ± 0.1	60.8 ^c^ ± 0.4
Syringic acid	8.4 ^a^ ± 0.0	149.8 ^d^ ± 0.4	29.8 ^b^ ± 0.1	49.4 ^b^ ± 0.0	42.0 ^b^ ± 0.2	82.3 ^c^ ± 0.6
*p*-hydroxybenzoic acid	72.8 ^b^ ± 0.0	49.9 ^b^ ± 0.1	89.3 ^b^ ± 0.3	148.1 ^c^ ± 0.1	14.0 ^a^ ± 0.1	27.4 ^a^ ± 0.2
Caffeic acid	0.2 ^a^ ± 0.0	3.1 ^b^ ± 0.0	2.3 ^b^ ± 0.0	12.3 ^d^ ± 0.0	1.2 ^a^ ± 0.0	7.4 ^e^ ± 0.0
Catechin	84.3 ^c^ ± 0.3	73.9 ^c^ ± 1.0	4.0 ^a^ ± 0.0	13.2 ^b^ ± 0.1	139.9 ^d^ ± 0,1	2.6 ^a^ ± 0.0
Epicatechin	1759.9 ^d^ ± 5.9	634.8 ^b^ ± 4.1	216.4^a^ ± 0.8	2085.5 ^e^ ± 1.0	1344.9 ^c^ ± 1.4	343.5 ^a^ ± 0.5
Quercetin	625.7 ^c^ ± 0.2	639.1 ^c^ ± 9.0	354.5 ^b^ ± 0.1	395.1 ^b^ ± 0.1	309.4 ^b^ ± 0.7	222.9 ^a^ ± 0.1
Rutin	324.4 ^a^ ± 0.5	481.0 ^b^ ± 0.3	1085.4 ^d^ ± 2.6	799.7 ^c^ ± 0.8	644.8 ^c^± 3.3	1764.7 ^e^ ± 31.7
Kaempferitrin	49.9 ^b^ ± 0.0	26.5 ^ab^ ± 0.0	441.3 ^d^ ± 0.2	13.7^a^ ± 0.1	9.7 ^a^ ± 0.0	180.4 ^c^ ± 2.5
Total [mg/ g dw extract]	4049.2 ^c^ ± 13.3	2466.9 ^a^ ± 4.2	2566.4 ^c^ ± 4.6	4903.5 ^d^ ± 6.2	3083.9 ^b^ ± 5.3	3561.3 ^bc^ ± 31.4

The mean values in the line marked with different small letters indicate the significance of differences (*p* ≤ 0.05). Abbreviations: dw: dried weight; MW: Magnum variety water extract; MaW: Marynka variety water extract; LW: Lubelski variety water extract; ME: Magnum variety ethanol extract; MaE: Marynka variety ethanol extract; LE: Lubelski variety ethanol extract.

**Table 3:** Correlation coefficients between AChE and BChE and estimated components in *Humulus lupulus* hops extracts.

Compound	AChE Activity	BChE Activity	DPPH (mmol Tx/g dw)	ABTS (mmol Tx/g dw)	ChA (%)
Chlorogenic acid	0.535 ^NS^	0.754 ^*^	0.225 ^NS^	0.811 ^*^	0.228 ^NS^
Ferulic acid	0.339 ^NS^	0.523 ^NS^	0.522 ^NS^	0.834^NS^	0.757^*^
Vanillic acid	0.621^*^	0.258 ^NS^	0.432 ^NS^	0.354 ^NS^	0.554 ^NS^
Gallic acid	0.654 ^NS^	0.265 ^NS^	0.865 ^*^	0.743 ^*^	0.335 ^NS^
*o*-coumaric acid	0.883 ^*^	0.056 ^NS^	0.387 ^NS^	0.433 ^NS^	0.432 ^NS^
*p*-coumaric acid	0.255 ^NS^	0.322 ^NS^	0.115 ^NS^	0.522^NS^	0.633 ^*^
Cinnamic acid	0.312 ^NS^	0.461 ^NS^	0.321 ^NS^	0.386 ^NS^	0.441 ^NS^
Syringic acid	0.308 ^NS^	0.225 ^NS^	0.532 ^NS^	0.276 ^NS^	0.654 ^NS^
*p*-hydroxybenzoic acid	0.297^NS^	0.821 ^*^	0.835 ^N*^	0.532^NS^	0.555 ^NS^
Caffeic acid	0.623 ^NS^	0.433 ^NS^	0.332 ^NS^	0.464 ^NS^	0.664 ^NS^
Catechin	0.411 ^NS^	0.609 ^NS^	0.376 ^NS^	0.742 ^*^	0.221 ^NS^
Epicatechin	0.854 ^*^	0.360 ^NS^	0.773 ^*^	0.228 ^NS^	0.667 ^*^
Quercetin	0.227 ^NS^	0.692 ^*^	0.321 ^NS^	0.799 ^*^	0.663 ^NS^
Rutin	0.471 ^NS^	0.783 ^*^	0.054 ^NS^	0.441 ^NS^	0.439 ^NS^
Kaempferitrin	0.694 *	0.210 ^NS^	0.354 ^NS^	0.115 ^NS^	0.330 ^NS^

Abbreviations: AChE: acetylcholinesterase; BChE: butyrylcholinesterase; DPPH: ability to scavenge 1,1-diphenyl-2-picrylhydrazyl radicals, ABTS^+^: ability to scavenge 2,2′azino-bis(3-ethylbenzothiazoline-6-sulphonic acid radicals; ChA: chelating activity; Tx: Trolox; dw: dried weight; * *p* ≤ 0.05; NS: statistically insignificant.

**Table 4:** Antioxidant activity of water and ethanol extracts of three hop varieties (Magnum, Marynka, Lubelski) by methods of ability to scavenge radicals in test with DPPH^·^ and ABTS^+.^

Sample	DPPH (mmol Tx/g dw)			DPPH EC 50 mg extract/mL)			ABTS (mmol Tx/g dw)			ABTS EC 50 (mg extract/mL)		
MW	4.75 ^e^	±	0.09	0.44 ^b^	±	0.12	1.32 ^a^	±	0.06	1.23 ^b^	±	0.10
MaW	4.37 ^d^	±	0.10	0.38 ^a^	±	0.08	1.43 ^a^	±	0.02	1.25 ^b^	±	0.03
LW	4.11 ^c^	±	0.06	0.31 ^a^	±	0.17	1.37 ^a^	±	0.05	1.26 ^b^	±	0.08
ME	4.12 ^c^	±	0.11	0.56 ^c^	±	0.06	2.33 ^b^	±	0.06	0.92 ^a^	±	0.08
MaE	3.50 ^a^	±	0.09	0.87 ^d^	±	0.12	2.43 ^b^	±	0.09	0.99 ^a^	±	0.04
LE	3.74 ^b^	±	0.11	0.98 ^e^	±	0.07	2.41 ^b^	±	0.13	0.93 ^a^	±	0.03
Quercetin	4.01	±	0.07	0.61 ^c^	±	0.03	1.65	±	0.11	1.05	±	0.06
Chlorogenic acid	4.65	±	0.03	0.77 ^c^	±	0.01	1.88	±	0.07	1.16	±	0.04

The mean values in the column marked with different small letters indicate the significance of differences (*p* ≤ 0.05). Abbreviations:DPPH: ability to scavenge 1,1-diphenyl-2-picrylhydrazyl DPPH radicals, ABTS^+^: ability to scavenge 2,2′azino-bis(3-ethylbenzothiazoline-6-sulphonic acid ABTS radicals, Tx: Trolox equivalent; dw: dried weight; MW: Magnum variety water extract; MaW: Marynka variety water extract; LW: Lubelski variety water extract; ME: Magnum variety ethanol extract; MaE: Marynka variety ethanol extract; LE: Lubelski variety ethanol extract.

**Table 5:** Inhibition growth area [mm] of selected microorganisms by tested hop extracts.

	Microorganism	MW	MaW	LW	ME	MaE	LE
1	*Propionibacterium acnes* ATTC 11827	9.0 ± 1.0	7.0 ± 1.0	6.0 ± 0.0	na	na	na
2	*Propionibacterium acnes* clinical isolates	7.0 ± 1.0	5.0 ± 0.0	5.0 ± 0.0	na	na	na
3	*Enterococcus faecium* ATCC 27270	4.0 ± 0.0	2.0 ± 0.0	2.0 ± 0.0	na	na	na
4	*Enterococcus faecium* clinical isolates	2.0 ± 0.0	1.0 ± 0.0	2.0 ± 0.0	na	na	na
5	*Staphylococcus aureus* ATCC 25923	39.0 ± 3.0	27.0 ± 2.0	18.0 ± 2.0	8.0 ± 1.0	4.0 ± 0.0	2.0 ± 0.0
6	*Staphylococcus aureus* clinical isolates	28.0 ± 2.0	22.0 ± 2.0	11.0 ± 1.0	4.0 ± 0.0	1.0 ± 0.0	na
7	*Staphylococcus epidermidis* ATCC 12228	34.0 ± 2.0	31.0 ± 2.0	12.0 ± 1.0	6.0 ± 1.0	5.0 ± 0.0	2.0 ± 0.0
8	*Staphylococcus epidermidis* clinical isolates	25.0 ± 2.0	26.0 ± 2.0	8.0 ± 1.0	3.0 ± 0.0	3.0 ± 0.0	1.0 ± 1.0
9	*Streptococcus salivarius* ATCC 13419	18.0 ± 2.0	15.0 ± 1.0	7.0 ± 1.0	5.0 ± 0.0	5.0 ± 0.0	na
10	*Streptococcus salivarius* clinical isolates	13.0 ± 2.0	11.0 ± 1.0	4.0 ± 0.0	2.0 ± 0.0	1.0 ± 0.0	na

Abbreviations: na: not active at the tested antimicrobials concentration; MW: Magnum variety water extract; MaW: Marynka variety water extract; LW: Lubelski variety water extract; ME: Magnum variety ethanol extract; MaE: Marynka variety ethanol extract; LE: Lubelski variety ethanol extract.
